# Comparative Evaluation of State-of-the-Art Semantic Segmentation Networks for Long-Term Landslide Map Production

**DOI:** 10.3390/s23229041

**Published:** 2023-11-08

**Authors:** Zekun Hu, Bangjin Yi, Hui Li, Cheng Zhong, Peng Gao, Jiaoqi Chen, Qianxiang Yao, Haojia Guo

**Affiliations:** 1 Badong National Observation and Research Station of Geohazards, China University of Geosciences, Wuhan 430074, China; huzekun@cug.edu.cn (Z.H.); cjq@cug.edu.cn (J.C.); yqx@cug.edu.cn (Q.Y.); ghj@cug.edu.cn (H.G.); 2 Three Gorges Research Center for Geo-hazard, Ministry of Education, China University of Geosciences, Wuhan 430074, China; 3 Yunnan Institute of Geological Science, Kunming 650051, China; bangjinyi@sina.com; 4 School of Earth Sciences, China University of Geosciences, Wuhan 430074, China; rslihui@cug.edu.cn; 5 Department of Earth and Ocean Sciences, University of North Carolina, Wilmington, NC 28403, USA; gaop@mailbox.sc.edu; 6 Department of Geography, University of South Carolina, Columbia, SC 29208, USA

**Keywords:** landslide inventory, machine learning, deep learning, transfer learning, U-Net3+

## Abstract

The production of long-term landslide maps (LAM) holds crucial importance in estimating landslide activity, vegetation disturbance, and regional stability. However, the availability of LAMs remains limited in many regions, despite the application of various machine-learning methods, deep-learning (DL) models, and ensemble strategies in landslide detection. While transfer learning is considered an effective approach to tackle this challenge, there has been limited exploration and comparison of the temporal transferability of state-of-the-art deep-learning models in the context of LAM production, leaving a significant gap in the research. In this study, an extensive series of tests was conducted to evaluate the temporal transferability of typical semantic segmentation models, specifically U-Net, U-Net 3+, and TransU-Net, using a 10-year landslide-inventory dataset located near the epicenter of the Wenchuan earthquake. The experiment results disclose the feasibility and limitations of implementing transfer-learning methods for LAM production, particularly when leveraging the power of U-Net 3+. Furthermore, following an assessment of the effects of varying data volumes, patch sizes, and time intervals, this study recommends appropriate settings for LAM production, emphasizing the balance between efficiency and production performance. The findings from this study can serve as a valuable reference for devising an efficient and reliable strategy for large-scale LAM production in landslide-prone regions.

## 1. Introduction

Landslides are among the most prevalent geological disasters worldwide, leading to substantial loss of human lives, infrastructure, agriculture, and industrial assets [1,2]. Detecting landslides is a fundamental and critical task in regional landslide investigation, as it facilitates informed decision making and risk mitigation by identifying the location, type, shape, and size of landslides [3,4]. Conducting field investigations for landslide detection is both time consuming and expensive. As a result, interpreting landslide information from remote-sensing images has become a viable and popular approach for generating landslide inventories over large areas.

While visual interpretation has been widely employed in many projects, it requires significant human resources and time to manually identify and verify landslides one by one, resulting in low efficiency and subjectivity [5,6]. Alternatively, a wide range of machine-learning methods, such as the analytic hierarchy process, decision tree, artificial neural network, support vector machine, and random forest, along with ensemble strategies, have been employed in landslide identification, leading to increasingly improved accuracy [4,7,8,9].

In recent years, deep learning has emerged as a promising approach for landslide detection, leveraging its ability to learn complex patterns and extract meaningful features from large datasets [8,10,11]. Compared to traditional machine-learning methods, convolutional neural network (CNN) architectures have shown superior accuracy in landslide detection using Rapid Eye images and 5 m spatial resolution DEM [10]. Notably, it has been verified that a residual network model (ResNet) outperformed general CNN architectures in terms of f1-scores. Following that, the U-Net model has been successfully employed to detect landslides in Rio de Janeiro, Brazil, and the Himalayan region, Nepal [9]. Following that, Semantic segmentation models, including U-Net and its variants, have been successfully applied to landslide detection in regions like Rio de Janeiro, Brazil, and the Himalayan region, Nepal [10,11,12]. In contrast to traditional deep convolutional neural network (DCNN) models, semantic segmentation results offer richer information about image components and finer details [11]. While numerous semantic segmentation models have emerged and continue to evolve, U-Net and its variations, such as U-net 3+ [13], ResU-Net [14], and TransU-Net [15], remain the most widely used models in current semantic segmentation research due to their superior performance.

Long-term landslide maps (LAM) document the occurrence, duration, recurrence time, and location of landslides. Indicators such as the cumulative occurrence rate, average persistence time, and recurrence rate can be derived from LAM, which play a crucial role in exploring landslide characteristics, such as sliding surface depth, geotechnical properties, and hydrogeological characteristics, and predicting landslide movements. Consequently, LAMs hold significant importance in unraveling the evolutionary process of landslides, capturing the spatial–temporal patterns of landslide movements, and evaluating and predicting regional stability [16,17]. Despite the successful application of various machine-learning and CNN models in landslide detection, most of them are focused on monotemporal mapping. As a result, LAMs remain scarce in many regions around the world [4,17].

The production of long-term landslide maps (LAM) poses a challenge, as it typically involves the collection of samples and training models on an annual basis or the manual assignment of features and thresholds by experts through a trial-and-error approach [16,17]. This process is time consuming and costly, especially for CNN models that require a large number of labeled image patches to fully utilize their advantages over traditional machine-learning algorithms Therefore, the development of efficient and cost-effective methods for producing LAMs is of great significance. In recent times, transfer-learning methods have emerged as solutions to expedite the training process, reduce costs, and address the challenge of acquiring extensive training datasets [18,19]. Its core concept is to apply knowledge acquired from source data in the training of target data. The three most common model-based deep transfer-learning methods include (i) Fine Tuning, (ii) Freezing CNN Layers, and (iii) Progressive Learning [20,21]. While various methods and practices have been reported [22], there remains limited exploration and comparison concerning the temporal transferability of state-of-the-art semantic segmentation models, especially in the context of LAM production.

In this study, an extensive series of tests was conducted to evaluate the temporal transferability of typical semantic segmentation models, specifically U-Net, U-Net 3+, and TransU-Net, using a 10-year landslide-inventory dataset located near the epicenter of the Wenchuan earthquake. This research aimed to achieve the following objectives: (1) Identify a model that demonstrates superior temporal transferability within this context and (2) assess the feasibility of generating LAMs using samples from limited years, which can serve as a benchmark for assessing the long-term stability of similar regions.

## 2. Materials and Methods

### 2.1. Study Area and Data

#### 2.1.1. Study Area

The earthquake that struck Wenchuan County in Sichuan Province, China, on 12 May 2008, stands as one of the most catastrophic seismic events in the country’s history, characterized by its magnitude, impact, and devastating loss of life. With extensive infrastructure damage, destruction of buildings, and disruption of industries, the earthquake caused significant economic losses amounting to billions of dollars. The human toll was also immense, with a tragic loss of over 69,000 lives [23,24,25].

**Figure 1:**
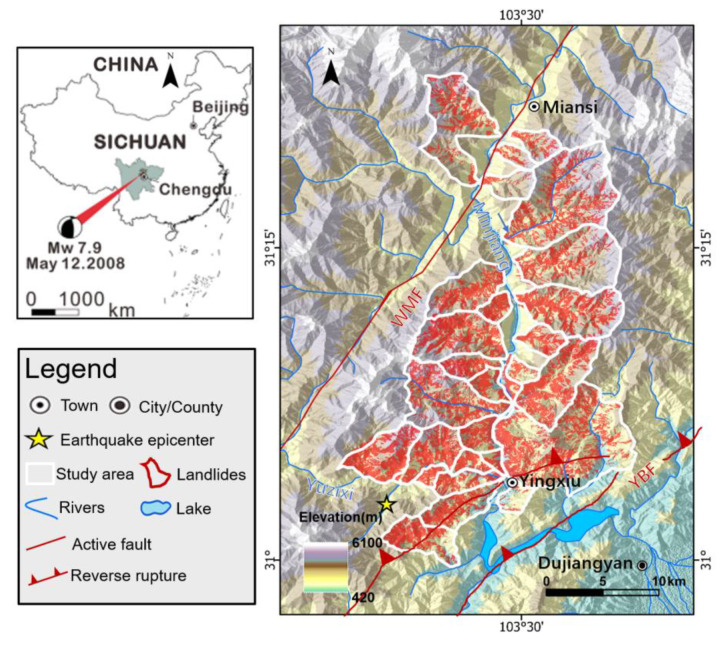
The study area.

The study area encompasses a relatively small portion, spanning over 471 km^2^ of the Longmen Mountains. It comprises 42 catchments stretching from the epicenter at Yingxiu town to Wenchuan County town (Figure 1). Situated within the Longmen Shan fold-and-thrust belt, the region has undergone extensive geological and tectonic activity, leading to the folding, faulting, and thrusting of rocks in the area [23,26,27]. The region showcases remarkable natural landscapes, characterized by deep gorges, dense forests, and steep slopes that rise rapidly from approximately 400 m above sea level to over 4000 m above sea level [25]. The study area experiences a subtropical highland climate, exhibiting distinct seasonal variations. The average annual temperature is 13 °C, and the mean annual precipitation is approximately 1250 mm, with the majority occurring during the summer months. Given the complex geology and ongoing tectonic processes in Wenchuan County, the region is susceptible to various geological hazards, including landslides, rockfalls, and debris flows. These hazards can be triggered by seismic activity, rainfall, or other factors, posing risks to the local population and infrastructure.

Following the earthquake, a staggering number of nearly 200,000 coseismic landslides were observed across a vast region exceeding 110,000 km^2^ [28]. Subsequently, the deposits of these coseismic events have been susceptible to reactivation triggered by rainfall, leading to catastrophic postseismic failures [25,29]. While several nearly complete inventories of the coseismic landslides have been compiled for the entire affected area, these inventories are predominantly monotemporal and, thus, inadequate for assessing the long-term landslide activity following the earthquake. In the specific study area, nearly 9000 coseismic landslides occurred and have been continuously monitored through the creation of a multitemporal inventory [30]. This comprehensive inventory can serve as a valuable reference dataset for testing and developing an effective temporal transfer-learning strategy, potentially contributing to the production of comprehensive LAMs for the entire earthquake-affected region.

#### 2.1.2. Remote-Sensing Data

Landsat imagery has proven to be a valuable tool for monitoring long-term land cover and land-use changes due to its relatively high resolution (30 m) and the availability of free long-term archives [1]. Numerous studies have demonstrated the suitability of Landsat images for regional landslide mapping tasks [6]. In recent years, Landsat imagery has been successfully utilized to detect and monitor long-term landslides in urban areas across Taiwan from 1998 to 2017 [17]. In our study area, it has been observed that 95% of the landslides are larger in size than a single Landsat pixel. This finding suggests that Landsat data is well suited for landslide detection purposes in the study area.

For the period from 2008 to 2018, Landsat TM (Thematic Mapper) and Landsat OLI (Operational Land Imager) scenes covering the study area were obtained from the USGS (United States Geological Survey) via the EarthExplorer website [31]. Specifically, the scenes from track No. 130/38 were downloaded for the growth seasons, which span from May to September. To ensure the quality of the data, pixels identified as “Cloud” with medium to high confidence levels in the Landsat Quality Assessment band were removed. This step aimed to eliminate cloud cover and ensure that the analysis is performed on cloud-free images. After removing clouds, all Landsat images for a specific year were merged to create the annual image through the median-value composite method. This composite image represents the central tendency of the pixel values for the entire time series, which can help reduce noise and outliers. This fusion technique produced complete cloudless annual images, which served as the basis for generating the complete long-term LAM. It is worth noting that Landsat ETM+ (Enhanced Thematic Mapper Plus) SLC-OFF (Scan Line Corrector Off) images were not included in the study. This decision was made due to the potential gaps in the data caused by the SLC-OFF issue, which could affect the overall quality of the long-term landslide maps.

#### 2.1.3. Reference Data

In the study area, a multitemporal landslide inventory has been produced by manually identifying landslides from high-resolution images [30], including aerial photo, SPOT, Worldview 2, Pleiades, and Landsat, for the years 2005, 2007, 2008, 2011, 2013, 2015, 2017, and 2018. In this study, we further conducted careful manual detection of landslides for the years 2009, 2010, 2014, and 2016 using high-resolution images in Google Earth Engine. These additional landslide detections were then combined with the existing multitemporal inventory, resulting in an almost continuous landslide inventory for the postearthquake years (as shown in Table 1). With the improved landslide inventory, comprehensive tests and detailed analysis of the models’ performances can be conducted.

Table 1 indicates that in the years preceding the earthquake, namely 2005 and 2007, a relatively small number of landslides were observed, with 133 (covering 0.72 km^2^) and 71 (covering 1.02 km^2^) landslides recorded, respectively. However, in the year 2008, following the earthquake, a significant increase was observed, with a total of 8924 coseismic landslides covering an area of 124.1 km^2^. Subsequently, from 2011 to 2018 (excluding 2012), varying numbers of landslides were recorded each year, ranging from 7617 to 10,136, and covering areas ranging from 88.1 km^2^ to 125.4 km^2^. Notably, studies indicate that there was a high occurrence of new and remobilized failures (such as landslides and debris flows) between 2008 and 2011, with a gradual decrease in the subsequent years since 2013 [30].

It is important to highlight that the number and area of detected landslides remained relatively consistent throughout the study period. This suggests that the activity of coseismic deposits and the long-term impacts of the earthquake have persisted and will likely continue. These findings emphasize the necessity of developing a long-term landslide inventory in areas significantly affected by major earthquakes. Further details about the inventory can be found in the literature [30].

### 2.2. Methods

To assess the temporal transferability of different models, we designed and tested four schemes, as illustrated in Figure 1. S1 was employed to evaluate the model’s performance in LAM production using the traditional yearly training approach, while S2 was utilized to assess the model’s temporal transferability in producing a 10-year LAM using one-year samples. Subsequently, in S3 and S4, we investigated the effects of patch size and time intervals on LAM production using the model that exhibited the best performance in S2. Throughout S1 and S2, various input data were utilized to determine the most suitable input for LAM production.

#### 2.2.1. Semantic Segmentation Models

Deep-learning models excel at automatically learning relevant feature representations from raw data, eliminating the need for manual feature engineering. This capability enables them to capture complex patterns and hierarchical representations from large datasets, resulting in improved performance and generalization. Deep-learning models have surpassed traditional machine-learning methods and achieved state-of-the-art results in various domains, such as image recognition, speech recognition, natural language processing, and game playing [8,10,11,32,33]. However, traditional convolutional neural networks (CNNs) face challenges in detecting fine details, outlines, and small entities within an image patch [9]. The use of pooling layers and down-sampling operations in CNNs can lead to a loss of spatial resolution, hindering the ability to capture fine-grained information.

**Figure 2:**
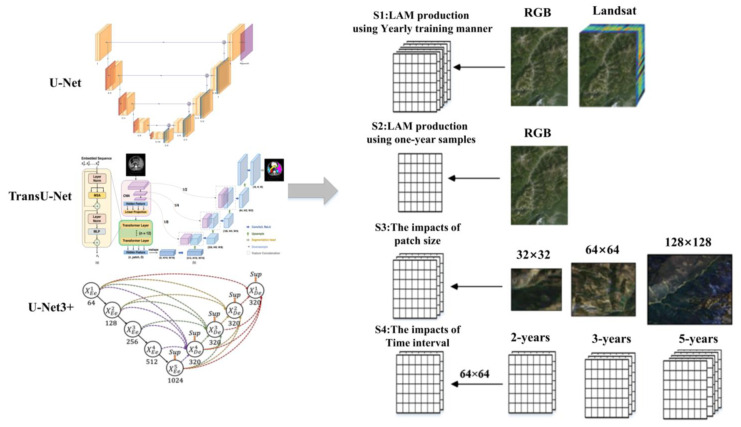
The schematic process flows.

Unlike traditional CNNs, the U-Net model was presented for extracting more precise details, consisting of an encoding path and a decoding path (as shown in Figure 2) [13,14,15,16,34]. The encoder path gradually reduces the spatial resolution while capturing high-level features through convolutional and pooling layers. The decoder path then upsamples the feature maps and recovers the spatial resolution using upsampling and transposed convolution layers. U-Net incorporates skip connections between the encoder and decoder paths, facilitating the direct propagation of information from the encoder to the decoder at the corresponding spatial resolutions. This mechanism helps preserve fine-grained details during the upsampling process, leading to improved segmentation accuracy. U-Net has proven to be a powerful architecture for image-segmentation tasks, especially in scenarios where precise localization and detailed segmentation are crucial, including landslide identification [10,11,34].

U-Net 3+ is an extension and improvement upon the original U-Net architecture. Instead of a single skip connection between the corresponding encoder and decoder layers, U-Net 3+ establishes multiple skip connections at different resolutions by introducing a nested and densely connected architecture (as shown in Figure 2) [13]. The dense skip connections in U-Net 3+ facilitate better feature extraction at multiple scales. By fusing features from different levels of the network, U-Net 3+ can capture both local and global contextual information, leading to more accurate segmentation results. The network can also better preserve and utilize spatial information during the upsampling process, resulting in sharper and more accurate segmentation boundaries. Overall, U-Net 3+ builds upon the strengths of U-Net while addressing its limitations, offering improved segmentation performance and more accurate results, particularly in scenarios involving complex and detailed images.

TransU-Net combines elements from Transformers and U-Net architectures to leverage the strengths of both (as shown in Figure 2) [15]. To improve the model’s ability to capture long-range dependencies and contextual information, TransU-Net incorporates Transformer modules. Transformers excel at capturing relationships between distant pixels in an image. TransU-Net also employs skip connections between the corresponding layers of the encoder and decoder, to preserve fine-grained details while processing high-level features. The integration of Transformers in the U-Net architecture allows TransU-Net to capture both spatial and contextual information effectively, making it suitable for tasks where understanding long-range dependencies is crucial.

#### 2.2.2. Test-Scheme Design

S1 was employed to assess the models’ performance in LAM production using the conventional supervised approach, where models were trained annually. This can also serve as a reference for the temporal transferability tests conducted in S2. In the test, models were trained and validated using samples collected within the same year. The samples were randomly divided into a training set (70%) and a validation set (30%). We conducted tests using three different band combinations, RGB and Landsat, as shown in Figure 2. The “RGB” combination utilized the red, green, and blue bands of Landsat imagery, enabling the evaluation of performance based on fundamental color information derived from the Landsat data. The “Landsat” combination encompassed all the spectral bands of Landsat imagery, excluding the thermal red band and Landsat OLI ultra blue, with the objective of capturing a wider range of information regarding the landslide surface. The aim of this test was to assess whether the integration of spectral information could enhance the detection performance.

S2 was designed to establish benchmarks for assessing the temporal transferability of semantic segmentation models. In this test, the models trained using samples from 2009 were utilized to generate landslide maps for another ten years. Compared to the previous evaluation [17], the time interval in this scheme is longer, and the evaluation metrics are more comprehensive, extending beyond overall accuracy. By employing comprehensive metrics, this scheme provides a deeper understanding of the models’ temporal transferability in LAM production. If a model demonstrates strong temporal transferability, indicating its ability to achieve satisfactory detection accuracy for multiple years using training samples from only a few years, it can significantly reduce the cost and time required for LAM production. Three distinct band combinations were also included in the test, as illustrated in Figure 2.

In S3, we specifically evaluated the influence of different patch sizes, namely 32 × 32, 64 × 64, and 128 × 128, on LAM production. By examining these various patch sizes, our study aimed to identify the optimal patch size that maximizes the accuracy and effectiveness of the transfer-learning approach in LAM production.

In S4, we tested several time intervals (1, 2, 3, 5, and 10 years) to determine the most suitable interval for LAM production. These intervals were compared with the yearly training test (S1) and the 10-year transfer-learning test (S2) discussed in previous sections. By understanding the optimal time interval, researchers can effectively balance the trade off between sample collection efforts and the accuracy of landslide detection in long-term monitoring tasks.

#### 2.2.3. Modeling and Accuracy Assessment

In the study area, the improved reference inventory was utilized to extract landslide sample images for training and validating the models. Each year, a total of 512 samples for the 32 × 32 size, 128 samples for the 64 × 64 size, and 32 samples for the 128 × 128 size, were collected. The semantic segmentation models were implemented using the Keras and TensorFlow libraries in the Python environment. It was trained and fine-tuned over 200 epochs, with a fixed learning rate of 0.001. The models utilized the Binary Cross Entropy loss function and the Adaptive Moment Estimation algorithm (Adam) as the optimization function. To prevent overfitting, the models’ progress was monitored during training, and the models were saved when the detection accuracy increased. This approach helps ensure that the models’ performance is not compromised by overfitting to the training data.

In the tests, the model’s performance in correctly detecting landslides was comprehensively assessed using several popular metrics, including overall accuracy ((TP+FN)/(TP+FN+FP+FN)), recall (TP/(TP+FN)), precision (TP/(TP+FP)), F1 score (2·Precision·Recall/(Precision+Recall)), and the known kappa coefficient. The overall accuracy (OA) measures the overall correctness of the model’s predictions. Recall indicates the model’s ability to correctly identify landslides from the reference dataset, thereby minimizing the number of missed landslides. Precision measures the model’s ability to minimize the number of false-positive predictions, i.e., points incorrectly classified as landslides. The F1 score is the harmonic mean of precision and recall, providing a balanced evaluation by considering both the precision and recall values. The Kappa coefficient takes into account the agreement that might be expected to occur by chance and provides a more robust measure of agreement than a simple percentage agreement. Here, TPs (true positives) indicate correctly detected landslides, TNs (true negatives) represent correctly identified nonlandslides, FNs (false negatives) indicate the reference landslides are mistakenly classified as nonlandslides, and FPs (false positive) represent objects incorrectly identified as landslides.

## 3. Results

### 3.1. LAM Production Using Yearly Training Manner

**Figure 3:**
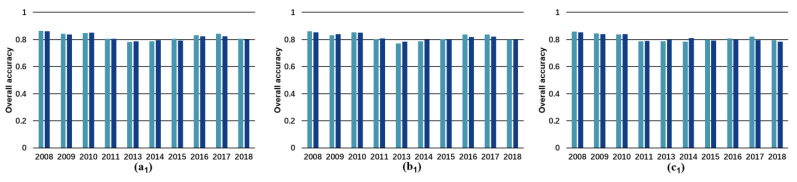

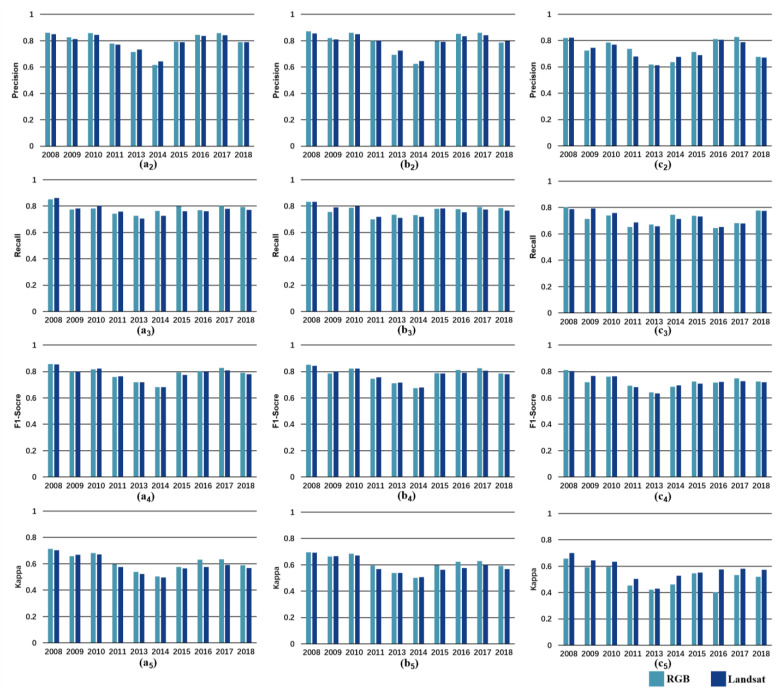
Performance of models with traditional yearly training (S1). The panels, (**a1**–**a5**), (**b1**–**b5**), and (**c1**–**c5**) depict the accuracy indicators (i.e., overall accuracy, precision, recall, F1 score, and kappa coefficient) for U-Net, U-Net 3+, and TransU-Net, respectively.

In Figure 3, we observe that U-Net 3+ achieved the highest performance, followed by U-Net and TransU-Net. It is worth noting that the differences among these models are not substantial, with most of their accuracy indicators surpassing 0.8. Interestingly, incorporating additional input data does not appear to significantly improve accuracy. In most cases, using RGB and all Landsat bands resulted in similar performance, whereas the inclusion of topographical features led to a decrease in accuracy. This can be attributed to the fact that RGB data provides ample contextual and spatial information for deep-learning models, while landslides triggered by earthquakes may not exhibit distinct topographical characteristics [25]. Comparatively, it can be observed that, although the OA achieved by the traditional machine-learning model (e.g., random forest) reaches 0.8, all other specific indicators are lower than 0.65, even when topographic features are included (Figure S1). This indicates that RF is not suitable for LAM production, even when employing the traditional yearly training approach. These tests also clearly demonstrate the strong dependence of the model’s performance on the type and quantity of input data. An interesting observation is that the detection accuracy of the random forest model experienced a significant decline in 2011, probably attributed to factors such as poor image quality or adverse air conditions during that year. In contrast, the accuracy of the DL model remained relatively stable during the same period, indicating its strong resilience to adverse conditions.

**Figure 4:**
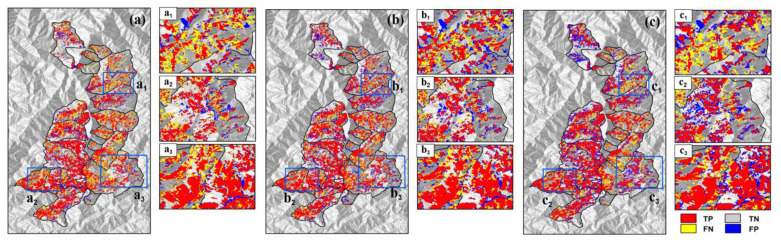
Examples of the S1 test results in 2009. Here, panels (**a**–**c**) demonstrate the results of U-Net, U-Net 3+, and TransU-Net, respectively. Panels (**a_1_**–**a_3_**) demonstrate the results of U-Net in the results visualization area. Panels (**b_1_**–**b_3_**) demonstrate the results of U-Net3+ in the results visualization area. Panels (**c_1_**–**c_3_**) demonstrate the results of TransU-Net in the results visualization area.

Figure 4 presents detailed results for the S1 test in 2009. In panel b and its subregions, it is apparent the detection result of U-Net3+ displays a significantly lower number of FN and FP pixels. Comparatively, other models exhibit a notable number of FN and FP pixels, indicating a higher rate of misclassifications. The U-Net3+ model demonstrates its capability to minimize both FN and FP errors, resulting in more reliable and accurate landslide-detection outcomes.

### 3.2. LAM Production Using One-Year Samples

**Figure 5:**
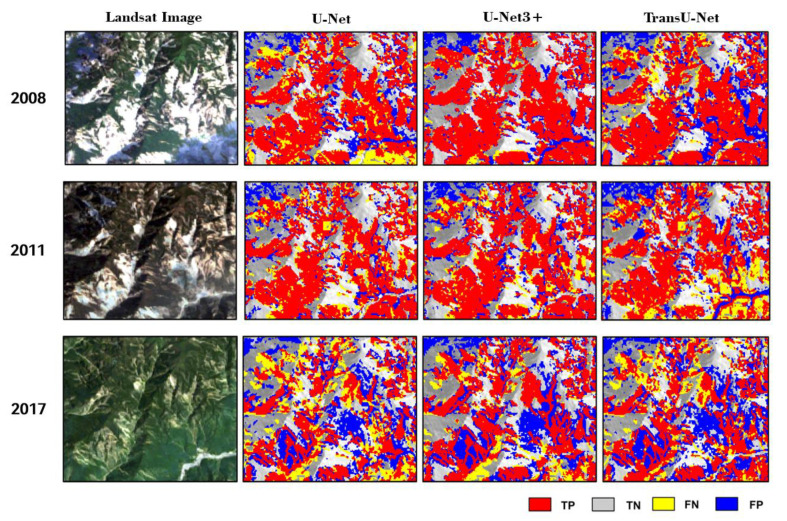
Examples from the S2 test results. Here, the first column displays the original images in the years 2008, 2011, and 2017, respectively. The second, third, and fourth columns depict the detection results obtained using U-Net, U-Net3+, and TransU-Net in results visualization area 2, respectively. This sample corresponds to subregion 1 in Figure 1.

The study conducted the S2 test to produce LAMs for a period of 10 years using samples collected from a single year (2009). Only the RGB channel was utilized in the test, as it demonstrated strong performance in previous experiments. The results of the test are presented in Table 2 and Figure 5.

Table 2 shows that the accuracy exhibits a noticeable decrease as the detection year moves further away from the training year (2009). In the initial years (2008, 2010, and 2011), coseismic landslides were relatively fresh and easier to identify, with surface features likely resembling those in the training samples from 2009. Consequently, higher performance could be achieved during these years. However, as time progresses, the overall accuracy gradually decreases from over 0.8 to around 0.7, while the F1-score decreases from approximately 0.8 to below 0.6. Comparatively, U-Net 3+ demonstrated the highest level of temporal transferability, followed by TransU-Net, and then U-Net. Figure 5 provides a visual representation of the S2 test results.

The gradual and slow decline in accuracy could be partly attributed to the evolution characteristics of landslides in the mountainous region under study. It is observed that most landslides undergo gradual natural changes, such as vegetation recovery or slope movement over the years [6], rather than experiencing dramatic changes caused by human activities or extreme events (Figure 5). Consequently, a model trained using one-year samples could achieve slightly lower performance in adjacent years, since the spectral and spatial differences between them are not significant. The test suggests that leveraging the temporal transferability of semantic segmentation models could serve as a dependable foundation for LAM production, provided that the time gap between the mapping year and the training year is not excessively long.

**Figure 6:**
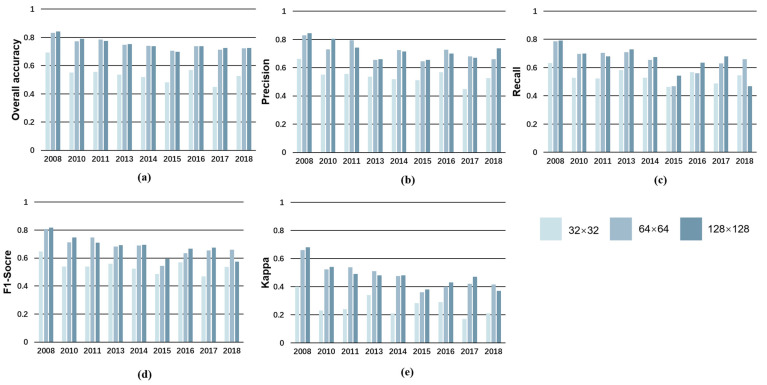
The results of the S3 test. Here, the panels, (**a**–**e**) depict the accuracy indicators (i.e., overall accuracy, precision, recall, F1 score, and kappa coefficient) for U-Net 3+ with RGB band combinations in different patch size.

Since U-Net3+ demonstrated superior performance in previous tests, it was employed in the S3 and S4 tests to unveil the impact of patch size and time intervals on LAM production. The results of the S3 test as Figure 6 indicate that there is a significant increase in detection accuracy, as the patch size increases from 32 × 32 to 64 × 64 for all years. Nevertheless, the enhancement almost plateaus when the patch size is expanded beyond 64 × 64 to 128 × 128. Considering the potential adverse effects on computation speed and training convergence, it is recommended to choose a patch size that strikes a balance between accuracy and efficiency. In this case, a patch size of 64 × 64 is considered an appropriate option, as it offers a significant increase in detection accuracy while avoiding the drawbacks associated with excessively large patch sizes.

**Figure 7:**
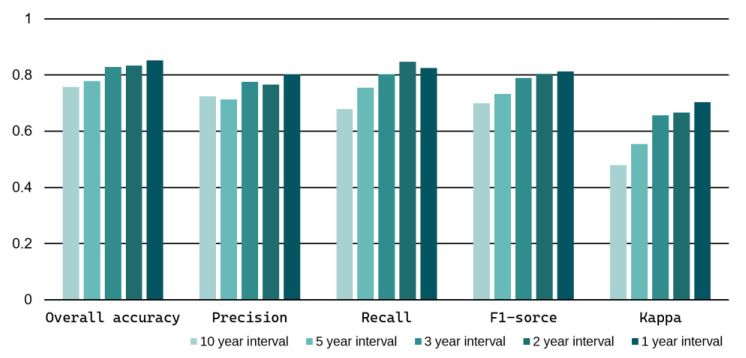
The results of S4 test. Here, the performances of traditional yearly training (S1) and transfer learning (S2) were also included as the “1-year interval” and the “10-years interval,” respectively.

It can be observed from Figure 7 that the detection accuracies show a negative correlation with the length of the time interval. The accuracies reach over 0.8 in the tests with short intervals (1 year and 2 years), while they decrease to about 0.7 in the tests with long intervals (5 years and 10 years). Obviously, the performance of the yearly training manner (S1) is greater than the 10-year transfer learning (S2). However, it is worth noting that the difference in accuracy between the short and long intervals is not substantial. This can be attributed to two factors: (1) the surface characteristics of most landslides in this mountainous region did not undergo substantial changes over the years, and (2) U-Net3+ demonstrates strong temporal transferability during these years. Comparatively, the three-year interval seems to be a favorable choice for achieving a balance between workload and accuracy in LAM production.

## 4. Discussion

While a LAM holds crucial significance in estimating the activity of landslides and assessing regional stability [35,36,37], its availability remains limited in most areas due to the high costs and time-consuming nature of large-scale LAM production. Despite the extensive use of various techniques, such as visual interpretation, machine-learning methods (including decision trees, artificial neural networks, support vector machines, and random forests), deep-learning models (such as ResNet, FCN, U-Net, etc.), and ensemble strategies [10,11,38] in landslide-detection studies, there is a paucity of research and production specifically focused on LAMs. As a result, the temporal transferability of these methods for LAM production has been inadequately explored. In earlier research, the construction of time-series NDVI (Normalized Difference Vegetation Index) curves using multiple sensors was employed to generate long-term landslide maps [16]. However, this approach heavily relied on expert domain knowledge and trial-and-error methods for process design and threshold assignment, resulting in limited adaptability and transferability. More recently, the random forest model was used to conduct long-term landslide detection and analysis in urbanized areas across Taiwan from 1998 to 2017 with Landsat images and nighttime light data [17]. However, it is important to note that the study did not utilize state-of-the-art CNN models and did not specifically discuss different transfer-learning strategies, including various models, data, patch sizes, and time intervals.

This paper conducted tests for producing LAMs using both traditional yearly training (S1) and transfer-learning (S2) approaches. S1 emphasizes the performance of semantic segmentation models compared to random forest in terms of accuracy and robustness in traditional yearly training, regardless of the input data. The temporal transferability and the models were checked through S2 when detecting landslides over a period of approximately 10 years using 1-year training samples. The gradual and slow decline of accuracy in S2 implies that leveraging the temporal transferability of those models could serve as a dependable foundation for LAM production when the time gap is not excessively long. It is important to note that, in this earthquake-affected high mountainous area, these models achieved detection accuracies (with F1-scores exceeding 0.8) similar to previous studies conducted in different regions [10,39]. This indicates that the models exhibit strong adaptability to various study areas and sample sizes. Such adaptability is highly advantageous for expediting and streamlining the production of LAMs, as it significantly reduces the required investment in terms of budget, labor force, and time.

Figure S1 shows the performance of the random forest was heavily influenced by the type and volume of the input data. This confirmed the positive impact of increasing input data in machine-learning landslide detection, which is consistent with previous research findings [38]. This is because random forest solely relies on the spectral features present in Landsat images, and the inclusion of DEM derivatives provides additional topographical information that aids the model’s understanding of landslides. On the other hand, for DL models, the addition of multispectral bands and topographical features did not significantly improve detection accuracy. This is because DL models are capable of extracting sufficient multilevel spatial information from visible images alone, making other features less influential in this case.

The slow decline in accuracy observed in the S2 test can be partly attributed to the specific characteristics of landslide evolution in the mountainous region. Previous research has indicated that the process of vegetation recovery to pre-earthquake levels can be a long-term phenomenon [25,40,41,42], and coseismic landslides may persist for approximately two decades under current climate conditions [24]. In this area, most landslides experienced gradual vegetation recovery or slope movement over the years due to factors, such as low precipitation, high evaporation, dry and barren soil, and limited human activities [25]. Furthermore, a separate study revealed that the vegetation recovery rate (VRR) on the surfaces of landslides within the entire earthquake-affected area was primarily influenced by elevation and slope, while climate factors, distance to the epicenter, and fault ruptures did not exhibit significant spatial variability in VRR [6]. These findings imply that the temporal transferability of landslide detection in this region may be less affected by the spatial variability of vegetation recovery.

In this study, Landsat images were chosen for regional long-term landslide detection, following the approach of previous works [17,43], owing to their freely accessible long-term archives and suitable resolution. While high-resolution images have the potential to detect smaller landslides [9,16], they are less suitable for building long-term archives due to issues of availability, usability, and cost. For example, in a previous study aiming to detect and monitor long-term landslide activity from 1986 to 2013, seven different optical images were combined, including SPOT 1 and 5, IRS1-C LISSIII, Landsat TM, and ETM+, ASTER, and RapidEye, to build a multitemporal satellite remote-sensing database [16]. The process of fusing different images entailed a substantial workload, and the uncertainties associated with using diverse image types were inevitable and challenging to estimate. Therefore, using Landsat archives as the primary data source for regional LAM production is a reasonable choice, especially considering that 95% of the landslides in the study area are larger than a Landsat pixel. Even after excluding smaller landslides, there were still thousands of landslides available, which provided sufficient data for training and validating the CNN model. It should be noted that the Sentinel-2 archives were not used in the study due to their limited coverage of the study years.

In the high mountainous area, the presence of clouds, haze, snow, or ice often poses challenges for image analysis. To mitigate this issue, the study utilized the Fmask algorithm [44] to identify and remove contaminated areas in the images. Subsequently, Landsat images captured during the growing seasons of each year were combined to generate annual cloud-free images, enabling a clearer analysis of the landscape. An additional limitation of the study was the unavailability of images for the year 2012. This was mainly due to the exclusion of ETM+ SLC-OFF images, which prevented the collection of samples and the validation of models specifically for that year. Consequently, a gap in the long-term tests and production exists, as the analysis could not be conducted for the specific period of 2012. However, it is important to note that this limitation does not significantly impact the overall assessment of long-term patterns and trends, as data from the other 10 years were still available and utilized in the study.

In the future, several tasks will be undertaken to enhance the performance and practicality of LAM production. First, the combination of Landsat and Sentinel-2 archives will be explored to create a more comprehensive and detailed LAM, by leveraging the strengths of both datasets. Secondly, the insights gained from the aforementioned tests will be applied to the entire earthquake-affected region after fine-tuning. By customizing the models and methodologies to suit specific areas, the aim is to streamline and expedite the process of generating large-scale LAMs. By implementing these improvements, the study expects to enhance the efficiency and effectiveness of LAM production, thereby providing valuable insights for estimating landslide activity and mitigating the risks associated with landslides.

## 5. Conclusions

In this study, an extensive series of tests was conducted to evaluate the temporal transferability of typical semantic segmentation models, specifically U-Net, U-Net 3+, and TransU-Net, using a 10-year landslide-inventory dataset located near the epicenter of the Wenchuan earthquake. The experimental results reveal both the potential and constraints of employing transfer-learning methods for LAM production, especially when capitalizing on the benefits of temporal transferability. Furthermore, the U-Net3+ model consistently exhibited superior accuracy, robustness, and temporal transferability in long-term landslide detection. Lastly, the study recommends that, when factoring in cost effectiveness, efficiency, and performance, it is prudent to operate with moderate data volumes, patch sizes, and time intervals. These findings are of the utmost importance in streamlining high-quality large-scale LAM production while minimizing budget, workforce, and time requirements.

## Figures and Tables

**Table 1:** The reference landslide inventory.

Year	2005	2007	2008	2009	2010	2011	2013	2014	2015	2016	2017	2018
Count	132	71	8924	8034	7617	9753	10,029	9644	10,124	9736	10,128	10,136
Area (km^2^)	0.72	1.02	124.13	117.2	113.02	88.11	110.79	118.58	125.43	125.03	125.08	123.85

**Table 2:** The results of the S2 test.

Year	U-Net					U-Net+++					TransU-Net				
	OA	Pre.	Rec.	F1.	Kap.	OA	Pre.	Rec.	F1.	Kap.	OA	Pre.	Rec.	F1.	Kap.
2008	0.81	0.82	0.73	0.77	0.58	0.84	0.84	0.79	0.82	0.68	0.80	0.76	0.83	0.79	0.63
2010	0.77	0.72	0.71	0.72	0.52	0.79	0.81	0.70	0.75	0.54	0.74	0.68	0.74	0.71	0.49
2011	0.78	0.78	0.72	0.75	0.54	0.77	0.74	0.68	0.71	0.49	0.76	0.74	0.80	0.77	0.58
2013	0.75	0.72	0.69	0.71	0.48	0.75	0.66	0.73	0.69	0.48	0.72	0.67	0.70	0.68	0.48
2014	0.74	0.69	0.70	0.69	0.46	0.74	0.71	0.68	0.69	0.48	0.73	0.66	0.58	0.62	0.37
2015	0.67	0.62	0.53	0.56	0.31	0.70	0.66	0.54	0.59	0.38	0.69	0.59	0.62	0.60	0.37
2016	0.73	0.62	0.75	0.68	0.43	0.74	0.70	0.64	0.67	0.43	0.70	0.62	0.71	0.66	0.42
2017	0.72	0.75	0.42	0.54	0.35	0.73	0.67	0.68	0.68	0.47	0.70	0.58	0.83	0.68	0.33
2018	0.69	0.65	0.36	0.47	0.23	0.72	0.74	0.47	0.57	0.37	0.70	0.59	0.56	0.57	0.28

OA, Pre., Rec., F1. and Kap. indicate overall accuracy, precision, recall, F1-score, and kappa coefficient, respectively.

## Data Availability

The original multitemporal landslide inventory released was downloaded from https://zenodo.org/record/1405490 (13 November 2018) [24]. The landslides of the years 2009, 2010, 2014, and 2016 identified by us are publicly available under Figshare [45]. Models were built and trained with the Keras and TensorFlow libraries in the Python environment. The codes for building random forest and U-Net are publicly available under Figshare [45].

## Supplementary Materials

The following supporting information can be downloaded at: https://www.mdpi.com/article/10.3390/s23229041/s1, Figure S1: Random Forest Performance in Traditional Yearly Training (S1). Panels a to d represent Overall Accuracy, Precision, Recall, and F1-Score, respectively, Figure S2: Examples of the Random Forest S1 test results in 2009, Figure S3: Examples from the S2 test results of Random Forest with RGB band. Here, the left column displays the original images in the years 2008, 2011, and 2017, while the right column showcases the corresponding results, Table S1: The results of the Random Forest S2 test.
