# Supplementary material for: Comparative Evaluation of State-of-the-Art Semantic Segmentation Networks for Long-Term Landslide Map Production
Source: Sensors (Basel). 2023 Nov 8;23(22):9041. doi: 10.3390/s23229041 (PMC10674776; doi:10.3390/s23229041)
Supplement: Supplementary file 1 [file sensors-23-09041-s001.zip › sensors-2671011-supplementary.pdf]

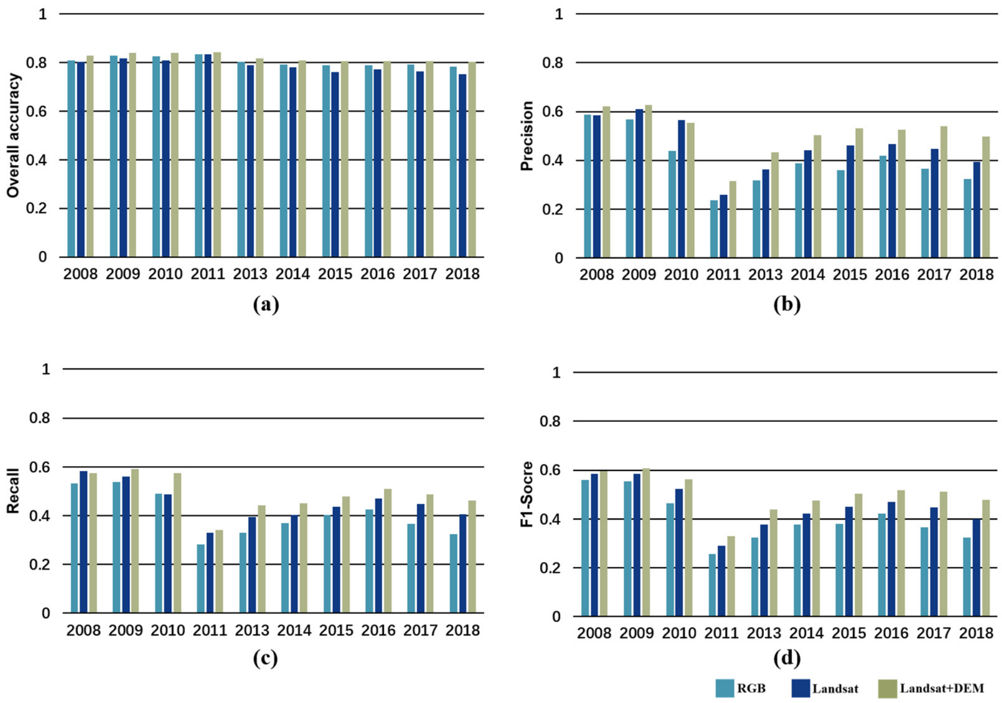

Figure S1. Random Forest Performance in Traditional Yearly Training (S1). Panels a to d represent Overall Accuracy, Precision, Recall, and F1-Score, respectively.

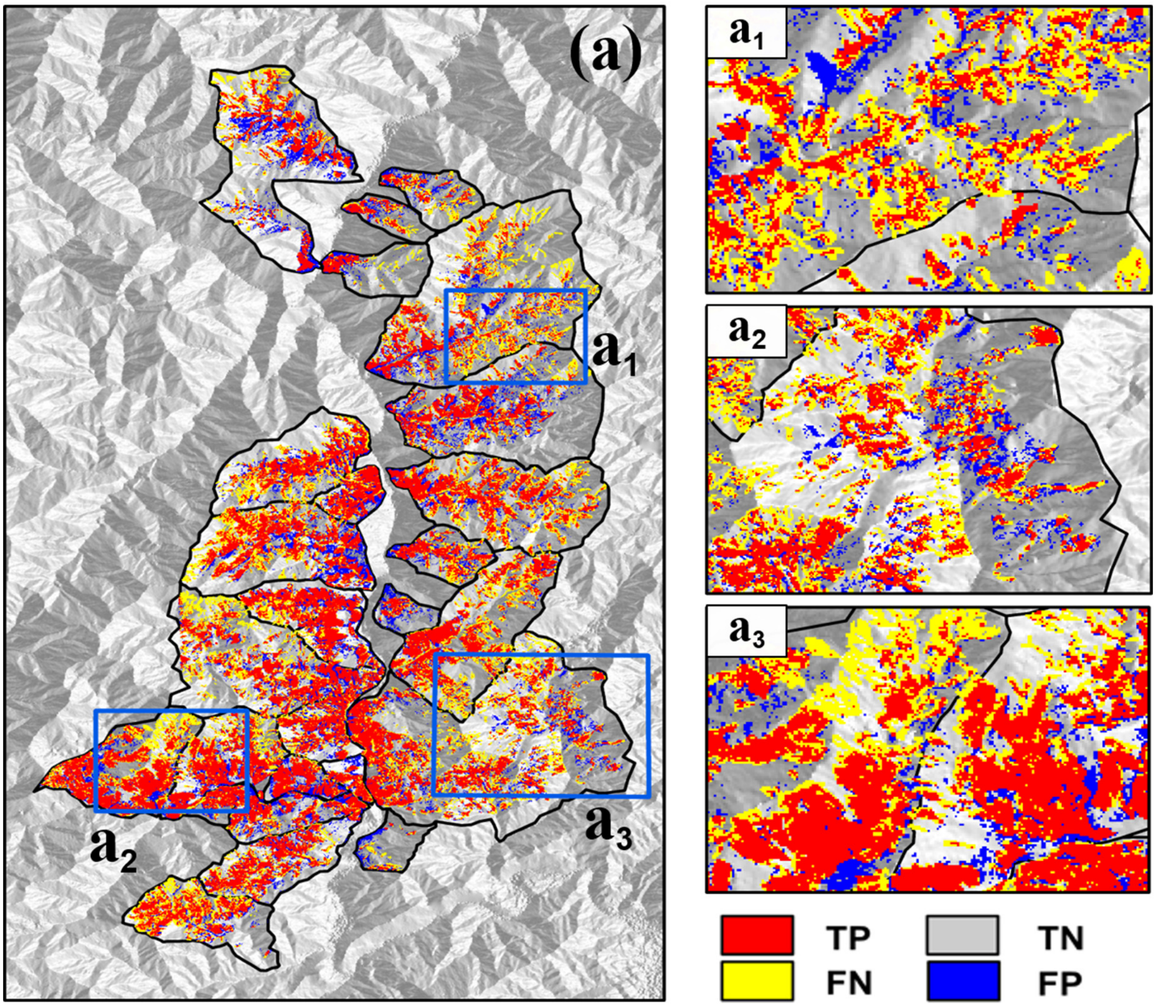

Figure S2. Examples of the Random Forest S1 test results in 2009.

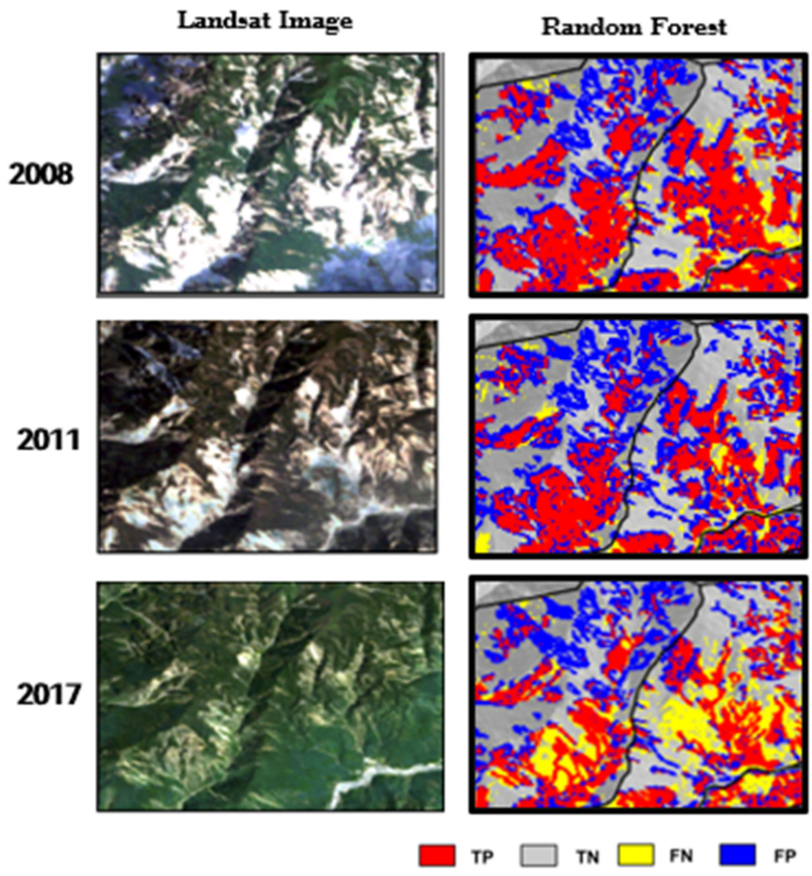

Figure S3. Examples from the S2 test results of Random Forest with RGB band. Here, the left column displays the original images in the years 2008, 2011, and 2017, while the right column showcases the corresponding results.

Table S1. The results of the Random Forest S2 test

Year	Random Forest			
	OA	Pre.	Rec.	F1.
2008	0.68	0.41	0.58	0.48
2009	0.65	0.41	0.51	0.46
2010	0.66	0.33	0.54	0.41
2011	0.66	0.4	0.54	0.46
2013	0.65	0.49	0.5	0.49
2014	0.63	0.44	0.41	0.43
2015	0.62	0.41	0.49	0.45
2016	0.59	0.44	0.51	0.47
2017	0.61	0.47	0.45	0.46
